# Modification of baseline status to improve breath tests performance

**DOI:** 10.1038/s41598-022-14210-0

**Published:** 2022-06-13

**Authors:** Estibaliz Alegre, Amaia Sandúa, Sofía Calleja, Sara Deza, Álvaro González

**Affiliations:** 1 Clínica Universidad de Navarra (Service of Biochemistry), Av. Pío XII 36, 31008 Pamplona, Spain; 2 IdiSNA, Navarra Institute for Health Research, Pamplona, Spain

**Keywords:** Gastroenterology, Diagnostic markers

## Abstract

Breath tests used to evaluate carbohydrates malabsorption require baseline H_2_ and CH_4_ levels as low as possible. Test cancellation is recommended when exceeding certain cut-offs (H_2_ ≥ 20 ppm and CH_4_ ≥ 10 ppm). Although following preparation protocols, many patients have baseline levels above those cut-offs. We investigated if light walking can reduce baseline H_2_ and CH_4_ levels. We retrospectively analyzed baseline H_2_ and CH_4_ levels from 1552 breath tests. Baseline levels (B1), especially in H_2_, were lower when obtained at later hours of the day. In those with baseline levels above cut-off, re-sampling (B2) after light walking for one hour, decreased H_2_ levels 8 ppm (Q1–Q3: 1–18 ppm), and 2 ppm (Q1–Q3: 0–3 ppm) for CH_4_. Consequently, 40% of tests with elevated B1 levels, presented B2 levels below mentioned cut-offs. Ten percent of tests considered negative when using B1 for calculations, turned positive when using B2 instead. All positive tests when using B1 values, remained elevated when using B2. Re-sampling after light walking for one hour could allow test performance in those with previous elevated baseline levels, avoiding diagnosis delays. Using the second sample for delta calculations identifies positive patients for malabsorption that would have been considered negative.

## Introduction

Malabsorption of carbohydrates can provoke several gastrointestinal symptoms including diarrhea, bloating, flatulence and abdominal pain due to intestinal fermentation of these non-absorbed sugars. Multiple pathologies can lead to this malabsorption, such as small intestine bacterial overgrowth (SIBO)^1^, lactase enzyme deficiency causing lactose malabsorption^2^, or the overload of fructose intestinal transporters (GLUT-2 and GLUT-5) causing fructose malabsorption^3^.

Currently, diagnosis of carbohydrates malabsorption is mainly based on breath tests. These null invasiveness tests consist on an oral load with the corresponding sugar and the monitoring of the altered absorption by measuring the fermentation gases (H_2_ and CH_4_) in exhaled air. An increase in H_2_ and/or CH_4_ levels reflects the intestinal fermentation of non-absorbed sugars. Rise in H_2_ or in CH_4_ levels depends on the type of anaerobic bacteria present. Although more prevalent intestinal bacteria produce H_2_, some patients present methanogenic bacteria that consume this H_2_ to produce CH_4._ As a consequence, both H_2_ and CH_4_ are recommended to be analyzed^4^.

To avoid false negative results, H_2_/CH_4_ baseline levels should be low^5,6^ and interpretation of elevated baseline levels is still uncertain^7^. We have previously shown that baseline high H_2_ levels are associated with more positive lactose breath test^8^. For this reason, patient preparation is required^7,9^, and it includes avoiding drugs such as antibiotic or laxatives, in the previous days, or procedures that could alter colonic microbiota such as colonoscopy. Besides, in the 24 h before the test, patient must follow a diet without fermentable fibers^10^, and the test must be performed in fasting state. In fasting conditions, baseline H_2_ levels are usually 7 ± 5 ppm^11^. However, elevated baseline levels are found in a significant number of patients due to different causes, such as SIBO, transgression in patient preparation, or constipation in the case of CH_4_^12,13^. For example, Kumar et al.^14^ showed that patients with irritable bowel syndrome have a baseline H_2_ concentration double than healthy population, while constipation correlate with baseline CH_4_ higher than 10 ppm^15^. In fact, some guidelines recommend not to continue the test if baseline levels exceed certain cut-offs ranging 15–20 ppm for H_2_^5,7^. This results in a delay of analytical results and diagnosis, and consequently in a detriment of patients. However, a recent survey stated that despite recommendations breath tests are still executed with a wide variety of conditions both in performance and in interpretation^16^. Although the requirement for baseline CH_4_ levels as low as possible also applies to CH4, guidelines do not clearly include a specific cut-off value^5,7^.

With the aim to decrease the number of high baseline H_2_ and/or CH_4_ levels detected during the performance of breath tests, we evaluated the effect of the time of procedure and a previous light walk.

## Results

### Basal breath test and patients characteristics

Median baseline levels of H_2_ were 6 ppm (Q1–Q3: 3–11 ppm) and 4 ppm (Q1–Q3: 2–10 ppm) for CH_4_. Considering the baseline cut-offs for H_2_ and CH_4_ of 20 and 10 ppm respectively, 11% of tests presented an elevated baseline H_2_ level and 25% an elevated baseline CH_4_ level. It should be noted that in 2% of tests, both H_2_ and CH_4_ were above their corresponding cut-offs (Table 1).

**Table 1:** Baseline H_2_ and CH_4_ levels according to age and sex of patients, indicated as median and interquartile range (Q1–Q3) is shown in a bracket.

		Median baseline levels ppm (Q1–Q3)		Baseline samples					
		H_2_	CH_4_	H_2_ high	CH_4_ high	H_2_ low/CH_4_ low	H_2_ high/CH_4_ low	H_2_ low/CH_4_ high	H_2_ high/CH_4_ high
Age	≥ 18	5 (3–10)	5 (2–11)	101 (8%)	340 (27%)	837 (67%)	77 (6%)	316 (25%)	24 (2%)
	< 18	9 (4–17) *	**4 (2–7) ~ **	65 * (22%)	48 * (16%)	196 (66%)	54 (18%)	37 (12%)	11 (4%)
Sex	Female	6 (3–12)	5 (3–11)	106 (11%)	252 (28%)	629 (64%)	80 (8%)	252 (26%)	26 (3%)
	Male	5 (3–10)	**4 (2–8) #**	60 (11%)	**110 # (19%)**	404 (72%)	51 (9%)	101 (18%)	9 (2%)
Total		6 (3–11)	4 (2–10)	166 (11%)	388 (25%)	1033 (67%)	131 (8%)	353 (23%)	35 (2%)

Baseline samples classification according to H_2_ and CH_4_ levels compared to their corresponding cut-offs. H_2_ high means ≥ 20 ppm, CH_4_ high means ≥ 10 ppm, H_2_ low means < 20 ppm and CH_4_ low means < 10 ppm. Comparing with patients above 18 years, * represents *p* < 0.01 and ~ represents *p* < 0.05. # represents *p* < 0.01 compared with female patients.

Regarding the age of patients, we observed higher baseline H_2_ levels in tests performed in underage patients (9 ppm; Q1–Q3: 4–17 ppm) than those in patients over 18 years (5 ppm; Q1–Q3: 3–10 ppm; *p* < 0.01; Table 1). On the contrary, in the case of CH_4_, levels were higher in adults (5 ppm; Q1–Q3: 2–11 ppm) than in underage patients (4 ppm; Q1–Q3: 2–7 ppm; *p* < 0.05). These differences were also reflected in the percentage of tests with baseline levels above the cut-off. In the case of H_2_, elevated baseline levels were observed in 22% of tests performed by underage patients, while this percentage decreased to 8% (*p* < 0.01) in those performed by older patients. Tests with elevated CH_4_ baseline levels were 27% of tests in patients above 18 years and only 16% of tests in underage patients (*p* < 0.01).

When considering the sex of patients, no differences were observed in baseline H_2_ levels or in the percentage of tests with baseline levels above the cut-off (Table 1). Also, female patients had slightly higher CH_4_ levels (5 ppm, Q1–Q3: 3–11 ppm) than male patients (4 ppm; Q1–Q3: 2–8 ppm; *p* < 0.01). Accordingly, we observed a higher percentage of tests with elevated baseline CH_4_ in female (28%) than in male patients (19%; *p* < 0.01).

### Basal breath test and time of the day

**Figure 1:**
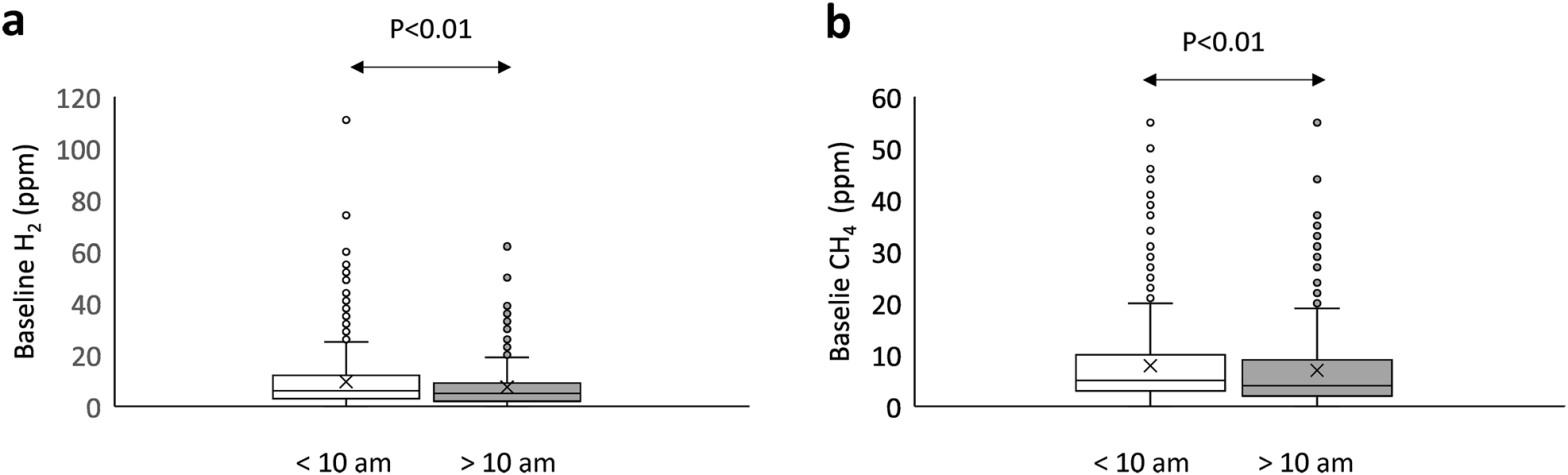
Baseline levels of H_2_ (**a**) and CH_4_ (**b**) according to sampling hour. Baseline levels are represented in Box-Whisker plots.

We also investigated the influence of the time of the day in baseline levels. We observed that baseline H_2_ levels changed with the hour of testing (Fig. 1a), being significantly higher (*p* < 0.01) in those performed before 10 am (6 ppm, Q1–Q3: 3–12 ppm) than those performed later (5 ppm, Q1–Q3: 2–9 ppm). Same occurred for CH_4_ levels (Fig. 1b), with higher values in tests performed in the early hours (5 ppm, Q1–Q3: 3–10 ppm) than in those performed after 10 am (4 ppm, Q1–Q3: 2–9 ppm).

**Figure 2:**
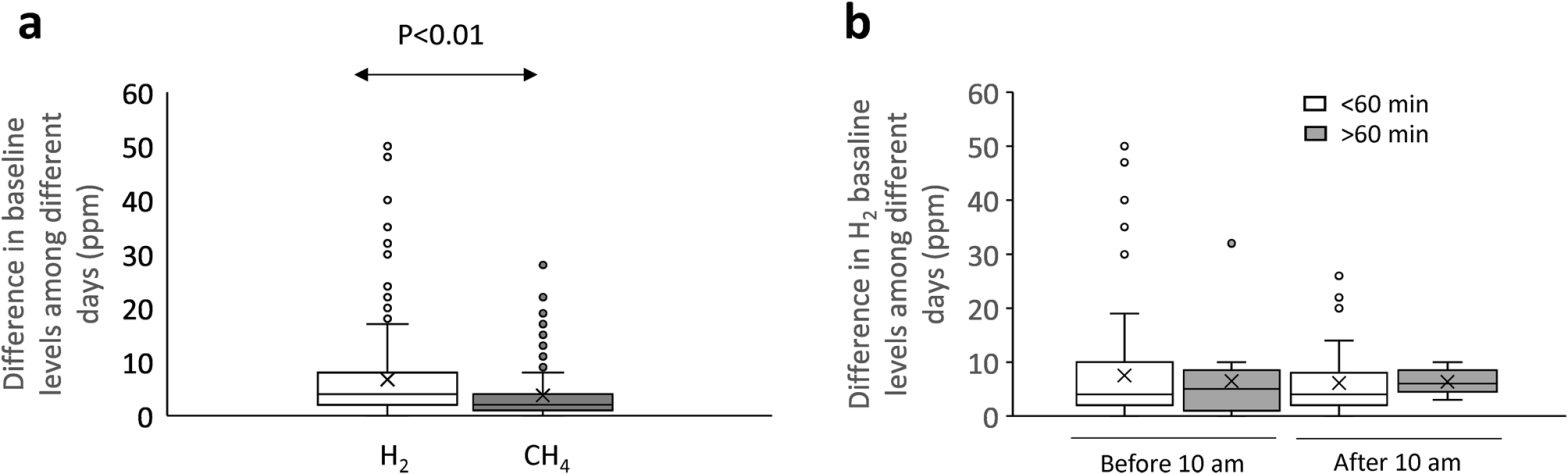
(**a**) Indifference in H_2_ and CH_4_ baseline levels between different days. (**b**) Difference in H_2_ baseline levels among different days depending on time of baseline sampling and whether sampling hours differ more than 60 min or not. Changes in baseline levels are represented in Box-Whisker plots.

As mentioned before, 240 patients performed multiple breath tests in different days. The range for baseline H_2_ levels in each patient among different days presented a median value of 4 ppm (Q1–Q3: 2–8 ppm), higher than 2 ppm observed for CH_4_ (Q1–Q3: 1–4 ppm; *p* < 0.01 Fig. 2a). The difference observed in H_2_ baseline levels did not depend on the hour of the day the sample was taken in or how distance were the different sampling hours (Fig. 2b). Median coefficient of variation of baseline levels among patients was 57% (Q1–Q3: 28–88%) for H_2_ and 35% (Q1–Q3: 16–71%) in the case of CH_4_.

This variation observed in baseline H_2_ levels obtained in different days, resulted in that 16% of patients presented an H_2_ baseline level below cut-off in one test and above that cut-off in another test performed in a different day (*p* < 0.01).

### Light walking and basal breath test

**Figure 3:**
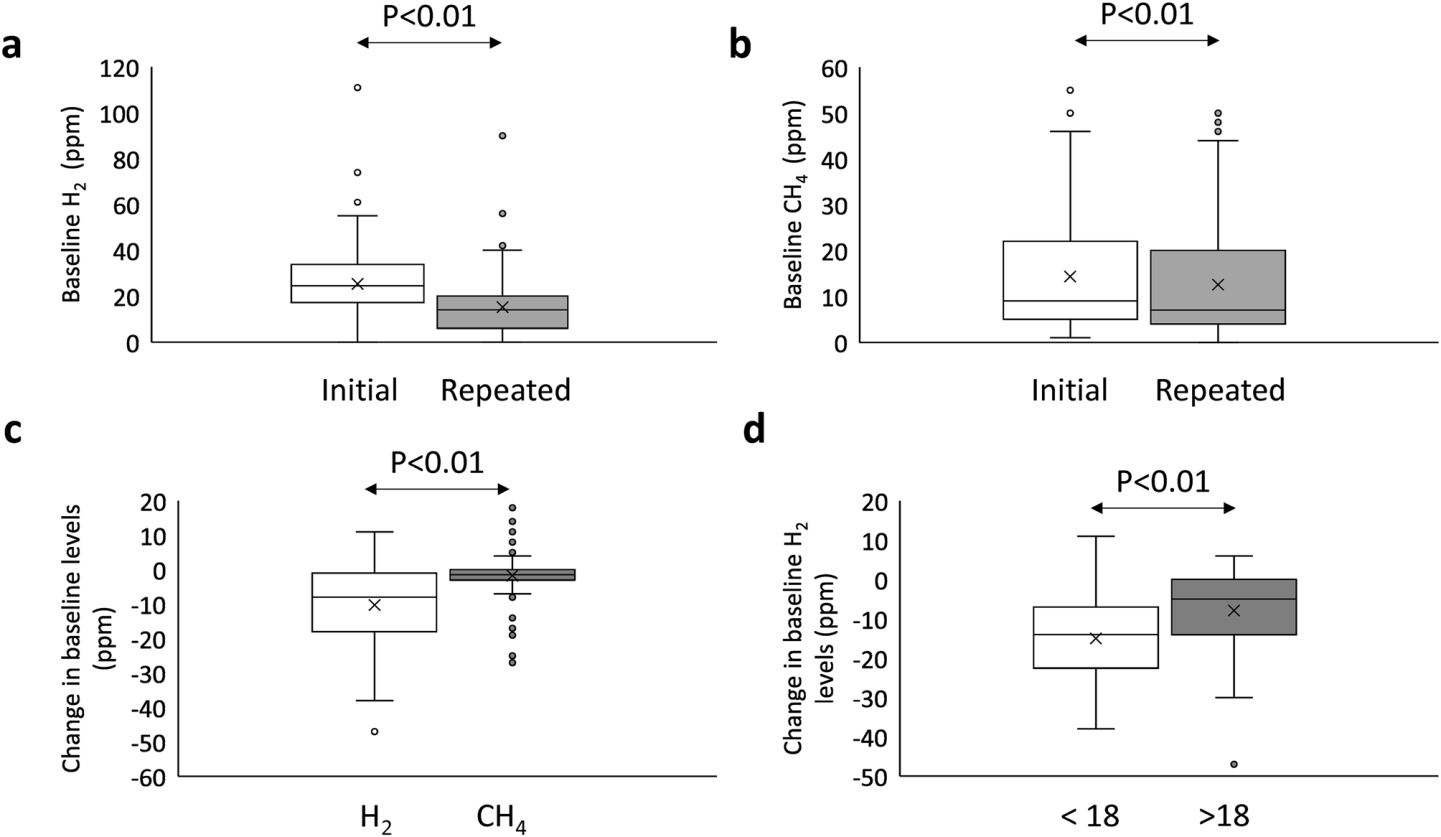
Initial and repeated baseline levels of H_2_ (**a**) and CH_4_ (**b**). (**c**) Range of change in H_2_ and CH_4_ levels between initial and repeated baseline samples. (**d**) Range of change in H_2_ levels according to patients’ age. Baseline levels are represented in Box-Whisker plots.

When baseline levels exceed established cut-offs, we obtained a new sample after 60 min of light walk. We performed this re-sampling in 158 tests, 90 (57%) due to high H_2_ levels, 39 (25%) due to high CH_4_ levels and 29 (18%) because of an elevation of both gases (Table 2). This re-sampling resulted in the decrease of baseline H_2_ levels from an initial median of 25 ppm (Q1–Q3: 19–34 ppm) to 15 ppm (Q1–Q3: 6–20 ppm) (Fig. 3a; *p* < 0.01). When considering CH_4_ levels, we observed a decrease from 9 (Q1–Q3: 5–22 ppm) to 7 ppm (Q1–Q3: 4–20 ppm) (Fig. 3b; *p* < 0.01). Median decrease in baseline H_2_ levels was 8 ppm (Q1–Q3: 1–18 ppm), higher than the observed in the case of CH_4_ (median: 2 ppm, Q1–Q3: 0–3 ppm, *p* < 0.01; Fig. 3c). If patient age was considered, significant differences were observed in H_2_ reduction (Fig. 3d), being much lower in the patients above 18 years (5 ppm; Q1–Q3: 0–14 ppm) than in underage patients (15 ppm; Q1–Q3: 7–23 ppm; *p* < 0.01).

**Table 2:** Baseline sample classification depending on initial and repeated baseline levels compared to the corresponding cut-offs (H_2_ ≥ 20 ppm; CH_4_ ≥ 10 ppm).

					Repeated baseline			
					H_2_ low CH_4_ low	H_2_ high CH_4_ low	H_2_ low CH_4_ high	H_2_ high CH_4_ high
Initial baseline	H_2_ high	*N* = 119	H_2_ high CH4 low	*N* = 90	58	30	0	2
			H_2_ high CH_4_ high	*N* = 29	5	3	14	7
	CH_4_ high	*N* = 68	H_2_ low CH_4_ high	*N* = 39	0	0	39	0

H_2_ high means ≥ 20 ppm, CH_4_ high means ≥ 10 ppm, H_2_ low means < 20 ppm and CH_4_ low means < 10 ppm.

These decreases in baseline levels after re-sampling resulted in that 67% of samples with initial high H_2_ levels, had a second baseline below 20 ppm cut-off (Table 2). In the case of CH_4_, only 12% decreased CH_4_ levels below 10 ppm cut-off after 1 h. As a whole, after baseline sample re-drawing, baseline levels below both cut-offs were observed in 40% of samples, 21% maintained elevated H_2_ levels, 33% elevated CH_4_ levels and 6% kept both H_2_ and CH_4_ levels elevated. These frequencies differed significantly from those observed in the initial measurement (*p* < 0.01).

### Breath test performance after baseline repetition

In 126 test with initial baseline (B1) levels above cut-off, after light walking and re-sampling (B2), tests continued with stimulus intake. If first baseline B1 was considered for delta calculations, H_2_ breath test was negative in 73% of the tests. However, 10% of these turned positive when delta was calculated with the repeated baseline B2 (Table 3). On the contrary, all the tests positive using B1, remained elevated when using B2 for calculations. This differences in classification reached statistical significance (*p* < 0.01).

**Table 3:** Performance of breath tests with baseline re-sampling.

			Positive test using the repeated baseline		*P*
			No	Yes	
H_2_	Positive tests using the first baseline	No	83	9	< 0.01
		Yes	0	34	
CH_4_	Positive tests using the first baseline	No	96	8	< 0.01
		Yes	6	16	

Tests were classified according to the presence of an increase in H_2_ or CH_4_ levels after stimulus intake, depending on whether first baseline of repeated baseline were considered for delta calculations. Increase in H_2_ levels was considered when ≥20 ppm and in CH_4_ levels when ≥10 ppm.

Related to CH_4_, there was an increase in 8 tests when considered the B2 but no increase when using B1 to calculate the delta. On the contrary, in 6 tests with increases related to B1, the increase disappeared when considering B2 for calculations. In this case, differences in classification were also statistical significant (*p* < 0.01).

## Discussion

To our knowledge, this is one of the first studies with larger sample size focused in baseline breath measurements. First of all, we observed the presence of methanogenic microbiota determined by the presence of CH_4_ in breath, in a high percentage of the patients, higher than the prevalence previously indicated^17,18^. Levitt et al.^18^ found that only 36% of patients presented CH_4_ levels higher than 1 ppm. Probably, this difference could be due to the different type of population analyzed. Prevalence of elevated CH_4_, baseline levels in our work was similar to that observed in a study of Harvie et al.^17^ who estimated a prevalence of 26% of high CH_4_ producers. However, they established 5 ppm as baseline cut-off. If using that value, the percentage of tests with elevated baseline CH_4_ in our population levels would have risen from 25 to 49%. This reinforces the indication of measuring both H_2_ and CH_4_ to avoid false negative results.

Our baseline H_2_ is similar to that reported previously^11^. Even when patients follow the indicated preparation, we have observed that baseline levels often exceed recommended cut-offs. When analyzing their demographic data, we found that those baseline levels were different between adult and underage patients. Since all samples included in the study had adequate CO_2_ levels, differences in H_2_ and CH_4_ levels cannot be attributed to an incorrect breath sampling technique in pediatric patients. Besides, H_2_ and CH_4_ performances are indeed opposite, which rules out contamination as the reason of the observed differences. On one hand, H_2_ baseline levels were higher in minor patients with a higher percentage of patients with baseline levels above the recommend cut-off. Le Neve et al.^19^ did not observe this association with patients’ age although their study was performed only in irritable bowel syndrome patients and not in suspected for carbohydrates malabsorption. Contrary to H_2_, fasting CH_4_ levels were higher in adult patients than in underage patient. This can be a consequence of the higher prevalence of constipation among adults^20^, which has been related to CH_4_ production, and other intestinal alterations such as diverticulosis^21^. We can reject the confounding effect of smoking in the results as patients were instructed not to smoke in the day of test performance and previous smoking effect in H_2_ and CH_4_ levels disappears after 10–15 min^22,23^.

Concerning time of day, we observed that H_2_ levels fluctuated more than CH_4_ levels, with higher levels earlier in the morning. To check if this was just a consequence of interindividual variability we focused in those patients with multiple tests in different days. We found that CH_4_ was much more stable than H_2_, contrary to that described by Jonderko et al.^24^. This could be due to sample size differences since our study comprised 240 patients compared to only 12 of Jonderko et al. In any case, H_2_ range across different days was reduced (although not significantly) when looking in those tests performed at same time of day (less than 60 min apart), suggesting that at least some of the variability observed can be attributed to the time of the day the test is performed. Shibata et al.^25^ found in healthy controls slightly higher levels (an increase of 2 ppm) of fasting H_2_ in the morning than before sleep the previous night. Related to this, we should consider that during the night there is a nocturnal hypoventilation that can cause these slightly elevated fasting levels of breath H_2_ produced by persisting fermentable substrates in the colon^26^.

Vigorous exercise is not recommended prior breath tests because it can alter ventilation rate and subsequently exhaled H_2_ and CH_4_ and its relation to CO_2_^27^. However, our indication is to go for a walk, light enough to not alter ventilation rate before and during the test. Even more, there was a minimum 5 min delay between patient returns and repeated sampling, and a visual inspection of potential hyperventilation. This light walk resulted in a reduction of baseline levels (mainly in H_2_ levels) that allowed to perform 40% of these tests. Interestingly, all tests in which only CH_4_ baseline levels were elevated, CH_4_ continued also elevated in the repeated baseline sample. Consequently, it seems unnecessary to perform the light walk and resampling when only CH_4_ is elevated in baseline samples.

The decrease in H_2_ levels after the light walk and the influence of time of day may be indeed related, as light activity could induce intestinal motility and feces stirring causing the release of preformed H_2_ trapped in the feces^28,29^. After one hour, this H_2_ would have been already released and the baseline levels would reflect more precisely the baseline state.

To date, it is not yet clear if elevated H_2_ baseline levels are related with diet transgressions, reflect SIBO presence^30,31^, or even pancreatic alterations such as exocrine insufficiency^32^ or pancreatic duct stenosis^33^. Although our protocol includes oral washing to avoid oral microbiota interference and patients are also questioned about potential diet transgressions that could affect breath performance, we have found that elevated baseline H_2_ levels were quite frequent (11%). However, they are critical since (i), when occurred, test cancelation is recommended and diagnosis is delayed and (ii), in the case of continuing, they can conceal a higher increase in H_2_ levels than those reflected in delta calculations, and thus lead to false negative results. In fact, we have showed that in those tests with high baseline levels and negative result, 10% of them would turn positive if the delta value were calculated with the repeated baseline level after light walking instead. However, all tests with high baseline levels and positive result would remained positive when repeated baseline was used to calculate the delta value.

In summary, breath status can be modified in patients by light walking, which reduces H_2_ and CH_4_ baseline levels. This allows tests continuation and avoids tests re-scheduling and diagnosis delays in a significant percentage of patients.

## Methods

### Patients

We retrospectively analyzed 1552 breath tests, from 1305 patients (Table 4), 756 (49%) fructose breath test and 796 (51%) lactose breath test. Of these, 232 (18%) performed two breath tests in different days and 8 (0.5%) performed three breath tests. Those tests were performed with a median of 9 days apart (Q1–Q3: 7–17). Female patients performed 64% of the tests studied. Median age was 36 years (Q1–Q3: 20–48). Patients younger than 18 years were considered underage, and performed 298 tests (19%). All methods were performed in accordance with the relevant guidelines and regulations^5^. The need of informed consent was waived and the study approved by Clínica Universidad de Navarra Ethic’s committee (project number 2020.222).

**Table 4:** Demographic characteristics of patients included in the study.

	*N*
Patients	1304
Age	36 (20–48)
Sex	987 female (64%)
Under-18 patients	298 (19%)
Age	9 (6–14)
Total breath test	1552
Fructose tests	756 (49%)
Lactose tests	796 (51%)
Tests per patient	
1	1064
2	232
3	8

Age is indicated as median and interquartile range (Q1–Q3) is shown in a bracket.

### Breath tests performance

Patients were required to perform the test in fasting and without having smoked that day. They had to accomplish a diet the previous day without fermentable fibers, lactose or fructose. Adherence to prescribed diet was checked before the beginning of the test. Fructose and lactose breath tests were not performed if patients had recently received antibiotics or laxatives or procedures such as colonoscopy. Prior to breath sampling, adults patients rinsed their mouths with a 10 mL of commercial oral antiseptic solution containing chlorhexidine. In underage patients rinses were performed with water. The stimulus were 25 g of fructose or lactose, respectively. In the case of pediatric patients, dose was adjusted to 1 g/kg with 25 g as the maximum dose.

Breath baseline samples and each 30 min for three hours after stimulus intake were obtained and 20 mL of them were analyzed for H_2_ and CH_4_ levels (ppm) in a Breath Tracker SC (Quintron, Milwaukee, USA). CO_2_ levels (in %) were also assayed simultaneously in the same analyzer to evaluate potential contamination of samples with ambient air^34^. If any sample presented CO_2_ < 2%, sample results were rejected and a new sample was obtained. The breath analyzer was daily calibrated and verified before processing any sample, using the gas calibrator provided by manufacturer. Our data indicate that imprecision and bias were respectively 3.1% and 4.7% for H_2_ measurement, and 3.4% and 4.1% for CH_4_.

According to guidelines, breath test lacks of reliability if baseline H_2_ exceed 20 ppm. If baseline samples (B1) rendered H_2_ above 20 ppm, a new baseline sample should be obtained. According to our own protocol and in order to achieve levels below that threshold, patients were indicated to go for a light walk outdoors for one hour with no goal in terms of distance. Although no specific cut-off is established for CH_4_ in guidelines, our protocol indicates the same procedure if baseline CH_4_ exceeds 10 ppm. The walk should be light enough to not provoke any stress, fatigue or hyperventilation. After arrival there were about 5 min of resting and a visual inspection of potential hyperventilation, before a new baseline sample (B2) was obtained. In the case that levels kept above the cut-off, breath test was cancelled or re-scheduled. Tests were considered positive when H_2_ or CH_4_ levels increased above baseline: 20 ppm in the case of H_2_ and 10 ppm in the case of CH_4_.

### Data analysis

Data were statistical analyzed with SPSS v20 software (IBM). Data are indicated as median an interquartile range (Q1–Q3). According to the normality tests performed (Shapiro–Wilk and Kolmogorov–Smirnov), both initial and repeated H_2_ and CH_4_ baseline levels followed non-normal distributions. Therefore, comparison between different patients were performed with Mann–Whitney U test whereas comparison between samples of the same patient was performed with Wilcoxon signed rank test. Frequencies were compared with χ^2^ test. *P* < 0.05 was considered as statistically significant.
